# Experimental Investigation on Machinability of α/β Titanium Alloys with Different Microstructures

**DOI:** 10.3390/ma16227157

**Published:** 2023-11-14

**Authors:** Shimaa El-Hadad, Ayman Elsayed, Bin Shi, Helmi Attia

**Affiliations:** 1 Central Metallurgical Research and Development Institute, Helwan P.O. Box 87, Egypt; ayman_elsayed_11@yahoo.com; 2 National Research Council, Montreal, QC H4P 2R2, Canada; bin.shi@cnrc-nrc.gc.ca (B.S.); or helmi.attia@mcgill.ca (H.A.); 3 Department of Mechanical Engineering, McGill University, Montreal, QC H3A 0C3, Canada

**Keywords:** alpha/beta titanium alloys, heat treatment, microstructure, machinability

## Abstract

In the current study, Ti-6Al-4V (Ti64) and Ti-6Al-7Nb (Ti67) alloys were prepared by vacuum arc melting. The produced samples were then subjected to different heat treatment regimes. The evolved microstructures and their corresponding hardness were investigated. Computerized drilling tests using TiAlN-coated high-speed steel bits were performed to assess the machinability of the prepared specimen regarding cutting force, tool wear, and thickness of the deformed layer. It was observed that Ti64 specimens that were water quenched from either α/β or β range contained martensitic phase. In Ti67, samples showed martensite only when water quenched from the β-phase range (1070 °C). Formation of martensite resulted in higher hardness and hence led to higher cutting forces and increased tool wear during the drilling process. Machined samples with higher hardness formed a thicker subsurface deformation area (white layer) and increased burr heights. Surface roughness in Ti64 workpieces was generally higher compared to Ti67 specimens. The coat of the drilling bit was partially attacked in the as-cast specimens, which was evident by elemental N on the machining chips. The machining tool deteriorated further by increasing the workpiece hardness through martensitic formation, where elements such as Cr, V, Fe, etc. that came from the tool steel were detected.

## 1. Introduction

Titanium and its alloys are superior engineering materials due to their unique combination of excellent resistance to corrosion, good fracture toughness, and of most importance, high specific strength. These unique properties have enabled several applications of titanium alloys in aerospace, power generation, chemical industries, and transportation [1]. However, being reactive materials with low thermal conductivity restricts broader applications of these alloys. Low thermal conductivity is a bad property in terms of machining because it increases the workpiece/tool temperature and adversely affects the tool life [2,3]. In addition, the ability of these alloys to keep their strength at elevated temperatures and to harden severely while being worked increases the machining force required and hence contributes to tool wear and minimizing of surface integrity [3,4]. Another concern is that the low modulus of elasticity of Ti-alloys is responsible for vibration and chattering during the machining process, which sometimes leads to workpiece deflection [5]. 

Extensive research has focused on improving the surface integrity of titanium alloys during the machining process [6,7,8,9]. Higher surface quality minimizes the cost of the post-machining process of ball burnishing, shot peening, etc. Therefore, reducing the surface roughness of the machined titanium components is considered a topic of current interest. Paulo [10] demonstrated that several trials have been performed to overcome the difficulties of machining titanium alloys, whether by altering the tool design [11], using different tool materials [12], implementing advanced hybrid processes (cryogenic machining [13], laser-assisted machining [14], vibration-assisted machining [15]), or working on the microstructure of titanium alloys to improve their machinability [16,17].

The microstructure is an essential factor contributing to the complication of the machining process of titanium alloys [18,19]. Pure titanium is fully α-phase, which is HCP structure at room temperature, while it changes its crystal structure to BCC, known as β-phase, upon heating to the beta transus temperature (~882 °C). This transition temperature is sensitive to any changes in the chemical composition [20]. Element groups known as α-stabilizers including aluminum, oxygen, and carbon raise the transition temperature and hence stabilize α-phase. Another group includes mainly vanadium, niobium, and molybdenum and reduces the transus temperature to stabilize β-phase. Fully α-alloys are characterized by ductility and weldability; however, they have limited formability due to their HCP structure. On the other hand, fully β alloys are distinguished by their high specific strength combined with good toughness. However, the small processing windows of this group of alloys limit their application. The two-phase (α/β) alloys exhibit an excellent combination of the properties of both types of alloys. Minor differences in the chemical composition of Ti-alloys can significantly affect their classification and hence their properties, including machinability [1,20].

Heat treatment of Ti-alloys can ultimately alter their microstructures [20,21,22] and, consequently, their machinability. Armendia et al. [21] showed that β annealing of Ti-6Al-4V alloy resulted in a coarse lamellar microstructure, which was translated to higher cutting forces and increased tool wear. Kosaka and Fox [23] also conducted several drilling tests on Ti64 and Ti54M alloys that were heat-treated with different regimes. It was observed that the most difficult-to-machine workpieces were those heat treated via β-annealing. 

Cast Ti-6Al-4V (Ti64) and Ti-6Al-7Nb (Ti67) alloys are two-phase titanium alloys, where α and β phases coexist in a so-called “Widmanstätten” cast microstructure with α-lamellae and β-layers. The alloys differ in the β-phase stabilizing element (V or Nb) and Ti67 has a finer microstructure than Ti64 [24]. In a recent study by Shehata et al. [25], it was found that the variations in alloy composition and microstructure between Ti-6Al-4V and Ti-6Al-7Nb alloys dictated different behavior in their machinability when cut using wire electric discharge machining (WEDM). Ti64 alloy, with the coarser microstructure, developed a thicker recast layer and higher surface roughness (Ra) than Ti67. These differences were also affected by the cutting conditions including the cutting mode and speed. The Ra of machined samples at cutting speed 50 µm/s under rough cut mode were 5.68 ± 0.44 and 4.52 ± 0.35 µm, for Ti64 and Ti7, respectively. They decreased to 1.37 ± 0.12 µm and 1.62 ± 0.18 µm at trim cut mode. Surface roughness followed the same behavior at 100 µm/s, in which Ra values for Ti67 alloy declined from 4.2 ± 0.34 µm at rough cut to 0.96 ± 0.08 µm at trim cut mode. 

Though some research was undertaken to compare the recently developed Ti67 alloy [26] to the commercial Ti64 alloy in terms of microstructure, mechanical properties [24], and biocompatibility [27], no work was performed to contrast their machinability using conventional machining processes (drilling, milling, etc.). Since these two alloys have some variations in their chemical compositions and exhibited different surfaces upon cutting using WEDM [25], they are also expected to show different machinability during the drilling process. In the current investigation, cast Ti64 and Ti67 alloy samples were prepared by the vacuum arc melting method and subjected to a series of heat treatments. The obtained microstructures were studied and the hardness was evaluated. Computerized drilling tests were performed to assess the machinability of the prepared samples regarding cutting force, tool wear, and thickness of the deformed layer. The machining results were then related to the processed microstructures of the different workpieces.

## 2. Experimental Work

### 2.1. Preparation of Cast Samples

**Figure 1:**
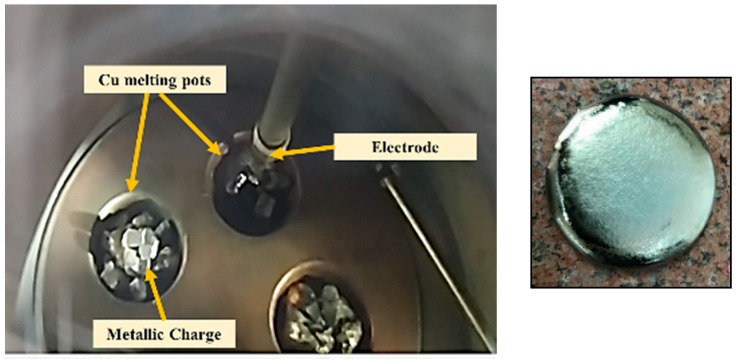
The melting vacuum chamber is used for melting titanium alloys and the as-cast titanium sample before cutting.

The investigation specimens used in the current study were prepared by remelting pieces of Ti-6Al-4V and Ti-6Al-7Nb alloys (provided by Baoji Xuhe Titanium Metal Co., Ltd., Baoji, China) in a vacuum arc furnace (model LHD 1250). The process starts by inserting pieces of each alloy in the melting chamber inside a copper-cooled mold. Figure 1 shows the melting process and the mold wherein the charge was inserted. This was followed by evacuating the melting chamber to avoid titanium reactivity and ensure a clean melting process. When the required vacuum level was reached, a high voltage was applied between the tungsten electrode and the copper mold. This resulted in an arc initiation, which melted the charge inside the mold. To guarantee homogeneity, electromagnetic stirring was used, and the samples were remelted several times until sound rod samples were obtained. The cast samples were finally cut into rectangles of (60 × 60 × 10 mm) using a wire-cutting machine (model RIJUN-FH 300). The chemical composition of the samples is shown below in Table 1. 

### 2.2. Differential Scanning Calorimetry

**Figure 2:**
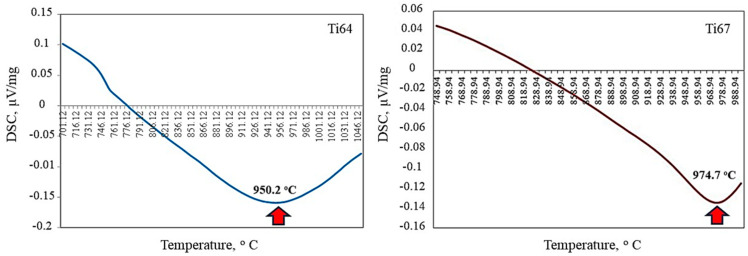
Results of DSC showing the transition temperatures of Ti64 and Ti67 alloys.

As an essential step before deciding the heat treatment scheme of titanium alloys; the transition temperatures of the two investigated alloys (Ti64 and Ti67) were determined via differential scanning calorimetry (DSC) [28]. A NETZSCH STA 409 instrument was used and a test sample of 55.3 mg weight was inserted into Al_2_O_3_ crucible in the DSC/TG pan under inert gas (He). The heating rate was 20/30 (K/min). The transition temperatures were found to be 950.2 and 974.7 for Ti64 and Ti67, respectively, as shown in Figure 2. Here, it is worth noting that the difference in transition temperatures of the two alloys is attributed to the type and amount of the beta-stabilizing element (V or Nb).

### 2.3. Heat Treatment 

**Figure 3:**
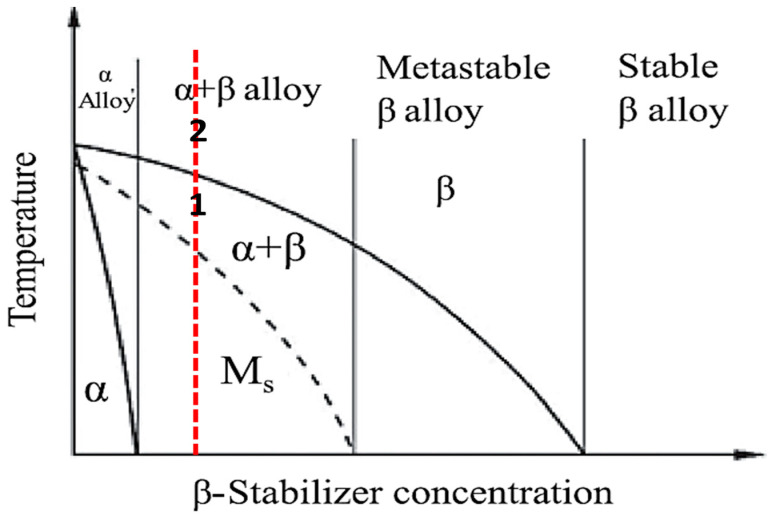
A section of the titanium binary phase diagram [20] indicating solution treatment temperature positions 1 (950 °C) and 2 (1070 °C). The dotted line refers to the approximate position of the investigated α/β alloys.

**Figure 4:**
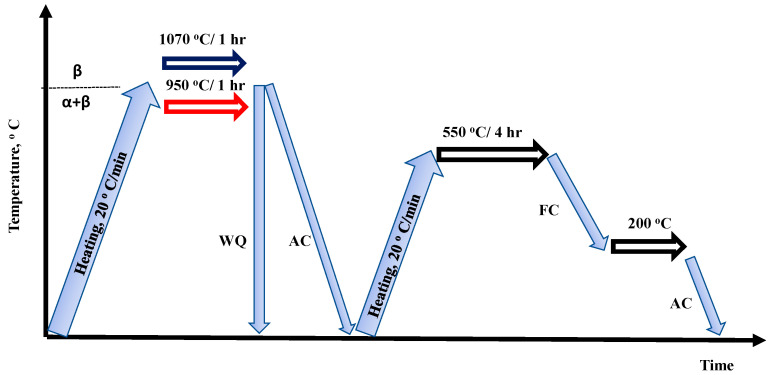
A schematic of the heat treatment cycles applied in the current work.

Heat treatment of titanium alloys is of primary importance in manipulating their microstructure and, hence, their mechanical properties. The solution treatment temperature is critical to the microstructure evolution after quenching [19,20]. Based on the transition temperatures shown in Figure 2, two treatment schemes were decided to solution-treat the samples from different phase fields to obtain varied microstructures. Figure 3 shows a section of the binary titanium phase diagram where the treatment positions (1 and 2) were roughly indicated. The workflow of heat treatment cycles is illustrated in Figure 4. The first step was solution treatment by heating the samples in a tube furnace to either 950 (α + β field/position 1) or 1070 °C (β-phase field/position 2) for 1 h at a heating rate of 20 °C per minute while maintaining a continuous flow of inert atmosphere (argon) to avoid titanium reactivity. 

The first group of the samples was water quenched (denoted as WQ) and the second group was cooled in air (AC). All the sample groups were then aged at 550 °C for four hours in the same furnace using the same heating rate of 20 °C per minute under an argon atmosphere. Finally, all the treated samples were left to cool in the furnace until 200 °C and taken out to air cool. After heat treatment, hardness was measured to provide a complete vision of all the workpieces before the machining tests. Sample codes and their definitions are listed in Table 2.

### 2.4. Drilling Tests

**Figure 5:**
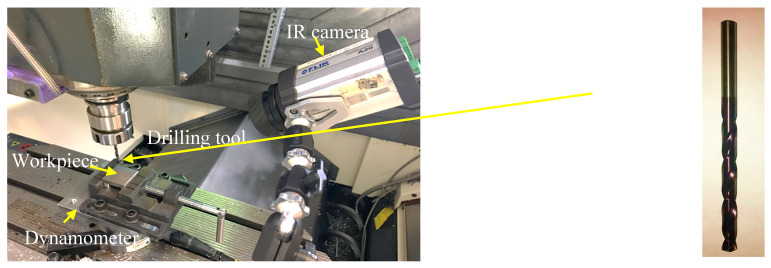
Machining experimental setup.

The cast and heat-treated specimens were ground to have a smooth surface for machining. The drilling tests were conducted on a DMU-100P machining center under dry-cutting conditions. Figure 5 shows the experimental setup. The drilling force and torque were measured using a four-component dynamometer (Kistler 9272, Winterthur, Switzerland). Preliminary drilling tests were performed to identify proper cutting parameters for drilling these materials. After a few trials based on the material hardness, drilling depth, and tool wear, the rotational speed of 800 rpm and feed rate of 0.1 mm/rev were selected and kept the same for all 10 samples. In each drilling test, a fresh tool (TiAlN-Coated High-Speed Steel Drill Bit, diameter 4.76 mm, point angle 135°) was used. 

**Figure 6:**
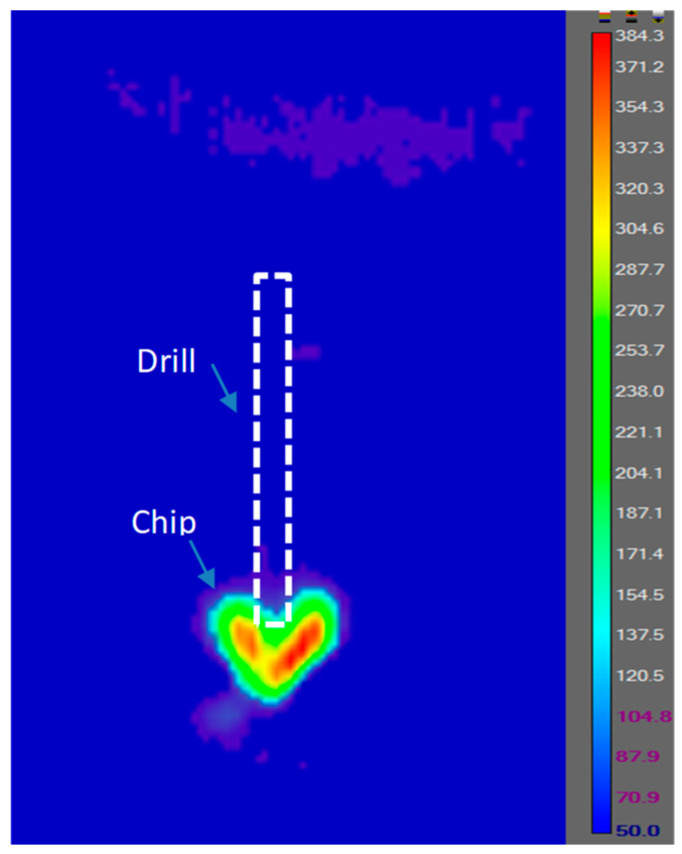
Position of temperature measurements.

An infrared camera (ThermoVision A20M, Saint-Jean-sur-Richelieu, QC, Canada) was used to monitor the temperatures of the workpiece, chip, and tool. To calibrate the emissivity, a small sample was heated from its bottom to a specific temperature using a torch. The top surface temperature was measured using a thermocouple welded on the top surface. At the same time, the temperature at the exact location was measured using the IR camera. Then, the reading from the IR camera was adjusted to the same value obtained by the thermocouple by changing the emissivity setting. Through this procedure, the emissivity of the workpiece was obtained. The emissivity values for the two types of samples were in the range 0.22–0.27. During the drilling processes, since the drill tip was hiding in the hole, the cutting edge was not exposed. Only the maximum temperature on the chips was extracted, as shown in Figure 6. 

**Figure 7:**
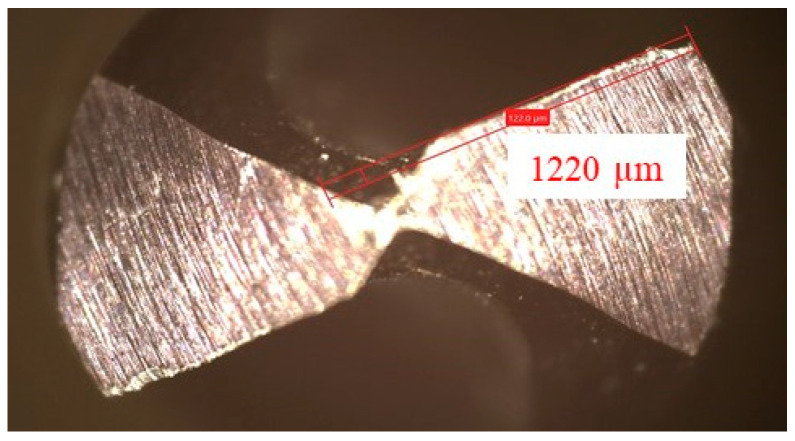
Measurements of tool wear.

After each test, tool wear was measured using a digital tool analyzer (ZEISS Smartzoom 5, Maple Grove, MN, USA), as shown in Figure 7. An average of the measured values was considered. The drilling forces and drilling torques were plotted and compared for the different specimens. The chips were collected after each test and kept for investigation. 

### 2.5. Sample Characterization

The as-cast and heat-treated samples were metallographically prepared according to the standard techniques and Kroll’s reagent of 10 mL (HF), 5 mL (HNO_3_), and 85 mL water was used for etching. The microstructure of the as-cast and heat-treated Ti-6Al-4V and Ti-6Al-7Nb alloys was investigated using an optical microscope (Zeiss). The machined specimens were sectioned along the drilled holes and the sub-machined surface features were detected using field emission microscopy (FEM). The machining chips collected from the different drilling tests were analyzed using energy dispersive X-ray (EDX) (Quanta FEG 250 model, Eindhoven, The Netherlands). The surface roughness of the sectioned holes was measured using a Talysurf Hobson (Series 2). 

## 3. Results and Discussion

### 3.1. Microstructure Investigation

**Figure 8:**
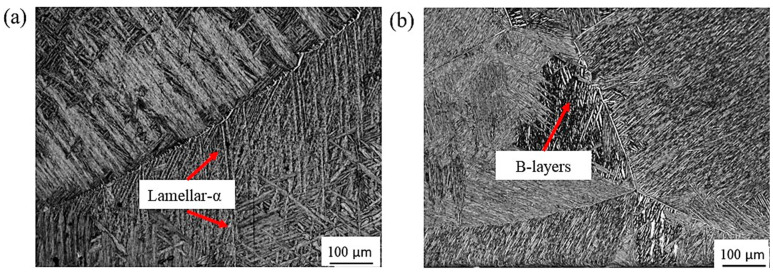
Optical micrographs of the as-cast (**a**) Ti64 and (**b**) Ti67 alloy samples.

Optical microstructures of the as-cast Ti64 and Ti67 alloys are introduced in Figure 8. Both of the alloys show typical α/β “Widmanstätten” cast microstructure with α-lamellae and β-layers. It is evident that alloy Ti67 containing Nb as a beta stabilizing element shows finer microstructure than Ti64.

In titanium (α + β) alloys, this two-phase microstructure is the equilibrium (room temperature) microstructure [19]. Upon solution treatment, β-phase has been reported to transform, whether partially or completely to either of the α-phase forms of martensite. The type of martensite (hexagonal (α′) or orthorhombic (α″)) depends on the solution treatment temperature and cooling rate [29]. Therefore, two different temperatures were applied and followed by two cooling rates (water and air) to have a variety of microstructures, through which the effects of microstructure on machinability could be assessed. 

**Figure 9:**
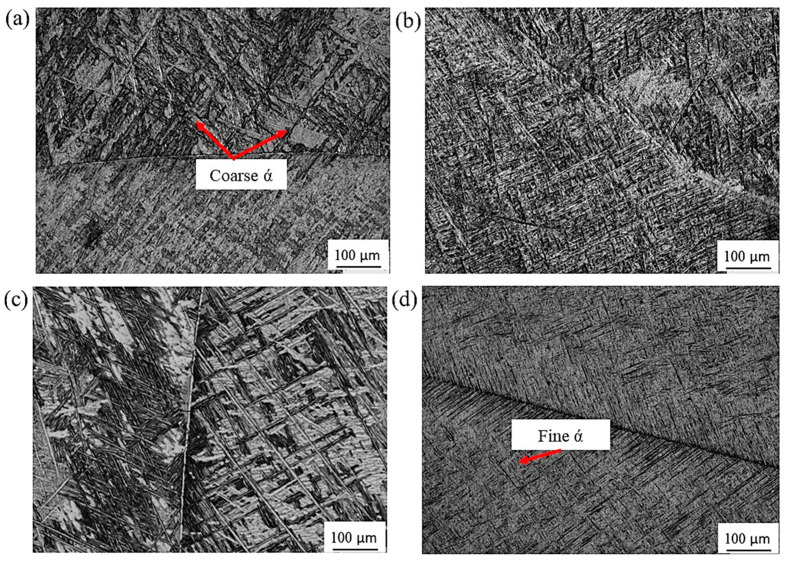
Optical micrographs of Ti64 alloy: (**a**) WQ950; (**b**) AC950; (**c**) WQ1070; (**d**) AC1070.

Figure 9 shows the optical micrographs of Ti64 alloy after solution treatment and aging. Solution treatment at 950 °C (α + β range) is known to produce α′ type of martensite in case of rapid cooling rate, which happens through a diffusionless process as reported earlier [20]. After aging, a structure of (α′+ primary α + β) obtained by WQ also contained fine secondary α as is evident from Figure 9a. On the other hand, AC from (α + β) field resulted in a microstructure containing (mainly primary α+ acicular α (transformed β) +β), where the thicker acicular α was observed, Figure 9b. This is in agreement with the review of Yadav and Saxena [29], where they attributed the formation of thicker α in the case of AC to the slower cooling rate. 

Solution treatment in the β-phase field followed by WQ at 1070 °C, and finally aging showed apparent martensitic transformation, where an almost complete martensitic α′ (α″) phase was obtained with some traces of α laths which increased in volume fraction due to martensite after the aging process (Figure 9c). According to Carrozza et al. [30], this type of microstructure (martensite) is mainly formed by applying cooling rates greater than 410 °C/S from a temperature sufficiently higher than the martensitic start temperature. It is notable that the acicular α (α′) is much finer in the case of cooling from the β-phase field (Figure 9c,d).

**Figure 10:**
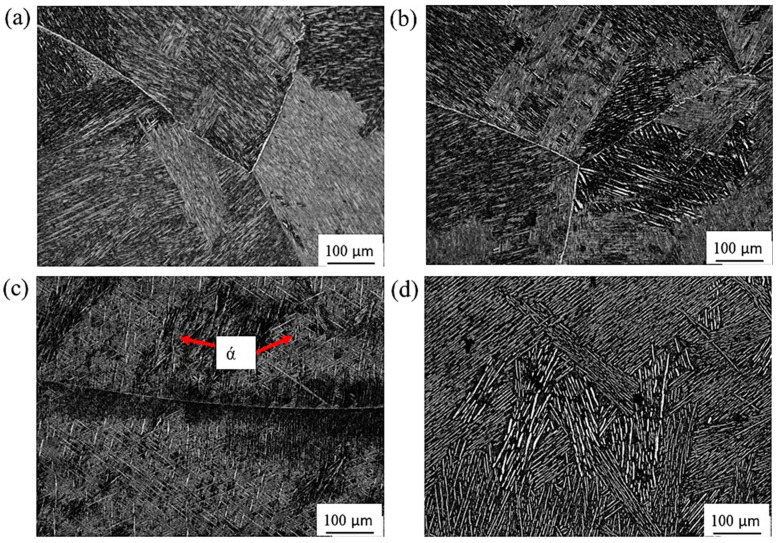
Optical micrographs of Ti67 alloy: (**a**) WQ950; (**b**) AC950; (**c**) WQ1070; (**d**) AC1070.

Figure 10 represents the micrographs of Ti67 alloy after heat treatment. Unlike in Ti64, there was no observed martensite upon cooling from 950 °C regardless of the quenching media. In Figure 10c, a microstructure where the martensite (α′) phase exists beside the bright α-phase and grey β-phase after WQ from 1070 °C. However, the amount of martensite was lower than in Ti64. In AC condition (AC 1070), no martensite was observed. Contrasting to the heat-treated Ti64, the martensite phase content achieved after WQ at 1070 °C was lower in Ti67, and no martensite was observed after AC from either α/β or β fields. This can be explained by the difference in the transition temperature (β_trans_) of the two alloys (DSC of Figure 2). The solution treatment temperature of 950 °C is just below β_trans_ of Ti64 (950.2 °C), while it is much lower than that of Ti67 (974.7 °C). Similarly, quenching from 1070 °C is well above β_trans_ of Ti64 compared to Ti67. This is in accordance with the work of Jovanovic et al. [31] and Reda et al. [32] on cast Ti64, where they concluded that increasing solution treatment temperature results in a larger volume fraction of martensite.

### 3.2. Cutting Forces and Tool Wear

**Figure 11:**
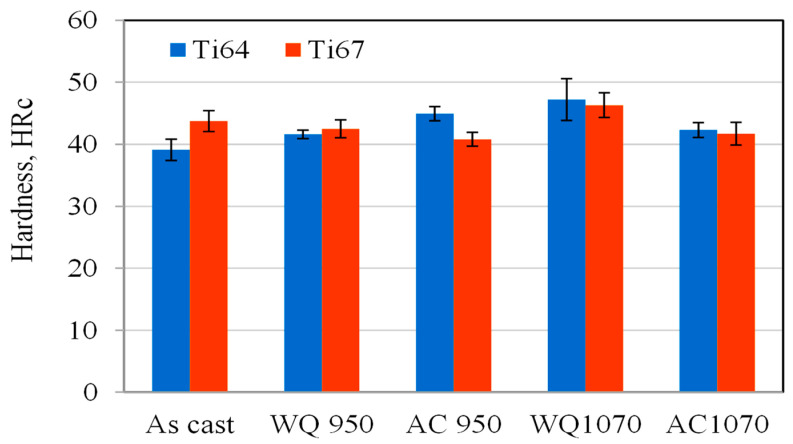
Hardness of cast and heat-treated samples.

The machinability of titanium alloys, including tool wear and cutting forces that are generated during the machining process, are both related to the workpiece hardness. In the current work, the hardness of all the specimens was measured before machining. Figure 11 represents the hardness values of the cast and heat-treated alloys. Generally, the hardness of the two-phase titanium alloys is strongly dependent on the martensite content after heat treatment. Martensite content in the case of Ti64 and Ti67 alloys is related to the solution treatment temperature and rate of cooling [1]. As the solution treatment temperature increases relative to the transition temperature and the specimens are quenched faster, martensite forms with a considerable amount, and hence the hardness increases accordingly. The hardness of as-cast Ti67 is known to be higher than that of Ti64 because of its finer grain size [26]. However, Ti64 showed a better response to heat treatment than Ti67, evidenced by the increase in hardness after cooling from the different phase fields. This can be due to the fact that the transition temperature for cast Ti64 is lower than that of cast Ti67 (Figure 2), while both of the alloys were treated at the same solution treatment temperature. Among all the investigated conditions, water quenching from 1070 °C resulted in the highest hardness due to the largest martensite content as explained in Section 3.1.

In the current drilling process, the materials are removed from the workpiece under the action of mechanical forces using a drill bit as the tool. It is very similar to the turning process. The drill bit plastically deforms the workpiece materials during the machining process [33]. The unwanted material removed, known as chips, will then flow through the flute of the drill bit to be removed from the drilled holes. This process is influenced by the feed rate, speed, bit geometry, cutting fluids, and the hardness of the workpiece material. The performance of the drilling process is assessed by measuring the material removal rate, the surface roughness of holes, tool wear rate, and burr formation. In the current work, tool wear, sub-machined surfaces layer, burr heights, and machining chips were all investigated and related to the processed microstructures.

**Figure 12:**
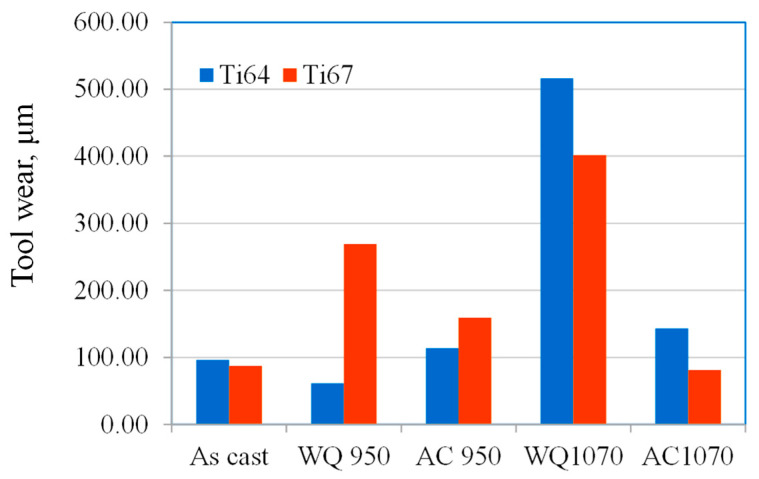

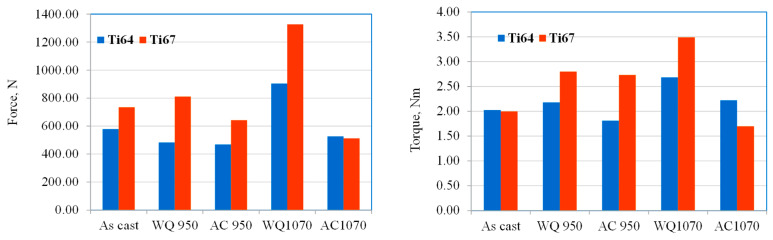
Tool wear; force and torque results of drilling tests versus workpiece condition.

Figure 12 presents the tool wear, force, and torque recordings versus the workpiece processing condition. It is clear here that the Ti64-WQ1070 specimen, which was treated by quenching from β-phase field and acquired the highest hardness, produced the largest tool wear, followed by Ti67-WQ1070. On the other hand, Ti67-WQ1070 exerted the highest force and torque values among all the tested workpieces. These results are in good agreement with Polishetty and Littlefair [34], where it was reported that the increase in Ti64 strength due to microstructure changes induced by heat treatment leads to deterioration of tool wear and can even cause a catastrophic tool failure in case of high speeds.

**Figure 13:**
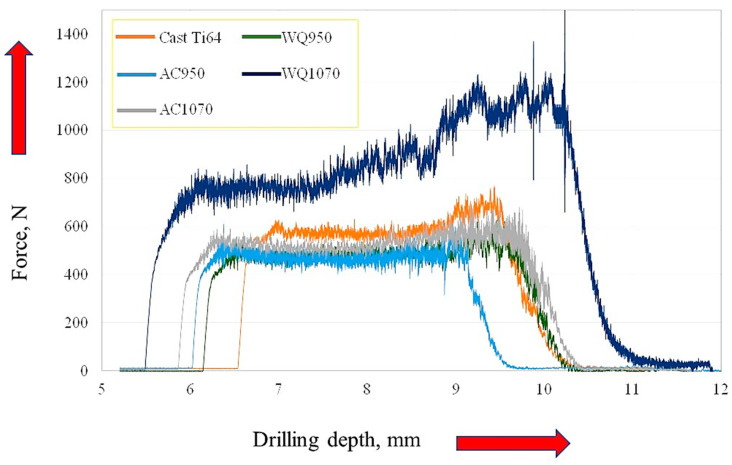
Typical cutting-force curves in surface drilling of Ti64 with different microstructures.

Figure 13 shows the typical force vs. drilling depth, including the three, reported drilling stages ((initial penetration (increasing force), feeding (stable force), and disengaging (decreasing force)). It is remarkable here that the force curve for Ti64-WQ1070 showed the least stable behavior during the feeding period. 

### 3.3. Drilling-Induced Sub-Surface Structures

**Figure 14:**
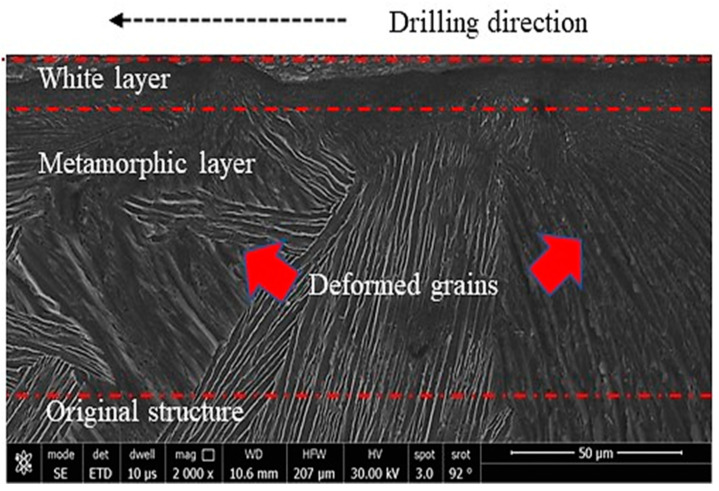
An example of FEM of subsurface structure showing the drilling induced layers.

**Figure 15:**
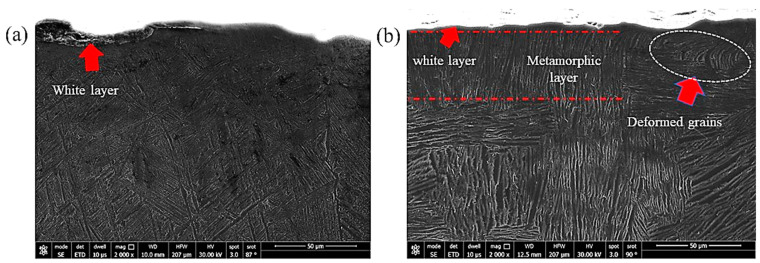
FEM showing the subsurface of the machined as-cast samples: (**a**) Ti64 and (**b**) Ti67.

In the case of machining, three distinct layers near the hole surface were described in the literature [35]. The first layer is the one that is squeezed by the action of the tool against the workpiece surface and is called the “white layer”; the second one is called the metamorphic layer, where the grains flow toward the machining direction; and the third layer is that containing the original microstructure. In the current experiments, a well-defined white layer, which can be better named the severely deformed layer, was observed. A clear example of the obtained subsurface microstructure is shown in Figure 14 and the different layers are demonstrated. It can be observed that this layer has no grains and it has a completely different structure. Figure 15 shows the sub-machined surface of the as-cast Ti64 and Ti67, respectively.

**Figure 16:**
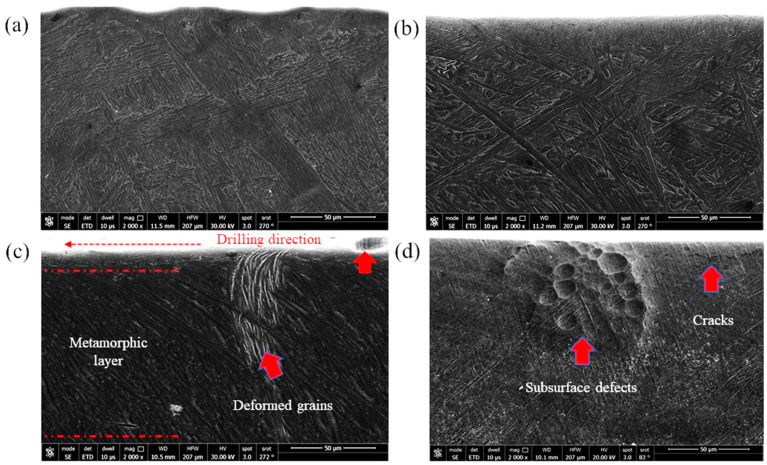
FEM showing the subsurface of the machined Ti64 samples: (**a**) WQ950; (**b**) AC950; (**c**) WQ1070; and (**d**) AC1070. Examples of machining-induced defects are indicated with arrows.

**Figure 17:**
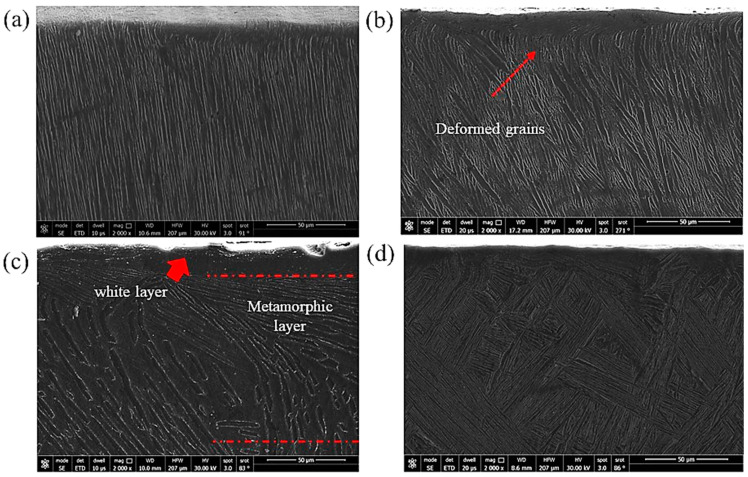
FEM of the subsurface of the machined Ti67 samples: (**a**) WQ950; (**b**) AC950; (**c**) WQ1070; and (**d**) AC1070.

Similar sub-machined surface layers were photographed using FEM for the heat-treated Ti64 and Ti67 and are shown in Figure 16 and Figure 17, respectively. The average thickness of the deformed layer was measured using these FEM micrographs, and all the measurements are listed in Table 3. 

Table 3 provides insight into the influence of the workpiece hardness on the deformed thickness. This is because of the shearing action produced during the drilling process, which forms such a layer. According to the work of Sato et al. [36] on a severely deformed Al-Ti alloy, the severely deformed layer that is induced by the shear force has a very special structure that possibly contains solid solutions, amorphous structures and nano-size phases/precipitates. Also, the thickness of this layer depends principally on the values of the applied load and alloy hardness. The current results (Table 3) are well in agreement with the reported work [36], where the thickness of the deformed layer showed apparent differences among the samples with varied microstructures/hardness. Moreover, the magnified subsurface FEM micrograph of Figure 14 shows a significantly fine structure in this severely deformed layer. 

Li et al. [37] observed that the formation of the white layer during the machining process occurs because of tearing the surface by plucking the particles and forming cracks that increase the material removal rate. Consequently, the grains change their morphology due to the movement under the shearing action of machining. As observed in the current investigation, superior structure refinement down to 1 micron size (with no grains) occurred in the white layer. In the next metamorphic layer, where the shearing influence is lower, a structure with oriented grains was observed following the direction of the applied force. Finally, when the force effect diminished, the microstructure returned to its original morphology. Figure 16 and Figure 17 show examples of the drilling-induced surface and subsurface defects (arrows). These machining defects have been reported as a source of fatigue failure. Therefore, the machining process variables should be optimized such that surface defects and cracks are minimized.

### 3.4. Burr Formation

Burr formation is a common phenomenon that occurs during the machining of Ti-alloys. Burr can be formed either at the entrance or the exit of the hole, and deburring may occur, leading to assembly problems [38]. In the current experiments, entry burrs were observed, and their heights changed with the alloy and/or microstructure of the workpiece. This kind of burr is commonly formed when the drilling bit comes in contact with the workpiece material, and the load is applied, the unremoved part of the material is compressed toward the circumference of the drilled holes and then raised, thus forming the entry burr [10].

**Figure 18:**
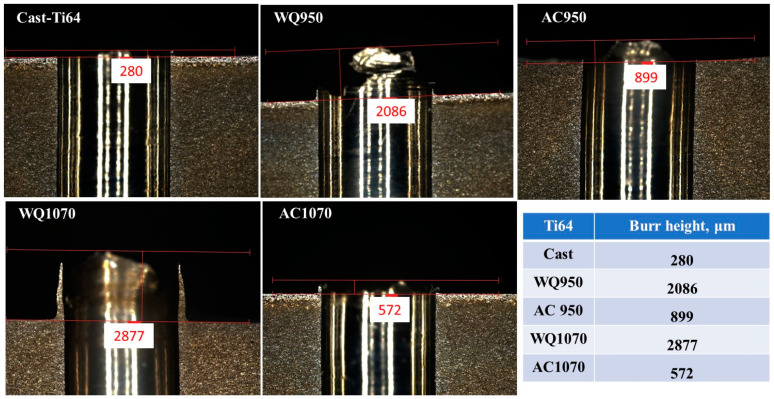
Burr height in Ti64.

**Figure 19:**
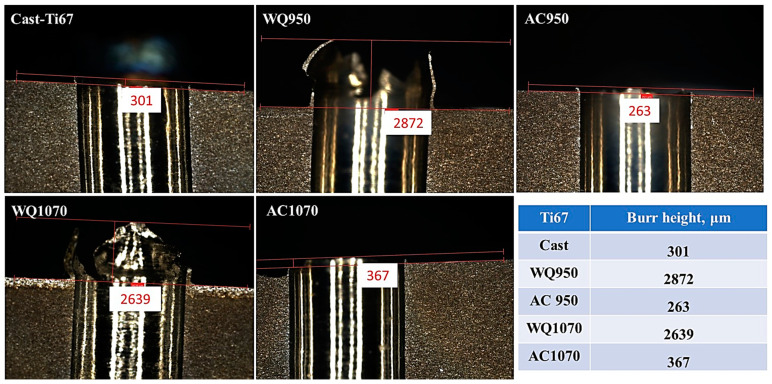
Burr formation in Ti67.

Figure 18 and Figure 19 show the burr height of the investigated Ti64 and Ti67 specimens. It has been reported that the twist drill type (which is used in the current study) produces a crown-type burr with a size that is larger than the crust burr generated by spiral drilling [39]. Here, it is worth mentioning that burr heights were generally related to the condition of the workpiece (microstructure and hardness). In the two tested alloys, water-quenched specimens with a significant amount of martensitic structure (Figure 9 and Figure 10) showed burrs exceeding a height of 2000 μ. On the other hand, the as-cast samples formed low burrs with ~250 ± 50 μ, and the air-cooled workpieces produced burrs with intermediate heights.

The current findings (force: Figure 12; and burr size: Figure 18 and Figure 19) agree with the work of Zhu et al. [40] on the drilling of Ti64. It was found that increasing the thrust force raises the test temperature and hence increases the burr height. This is because higher processing temperature favors plastic deformation of the workpiece material. 

### 3.5. Surface Roughness and Chip Characteristics

**Figure 20:**
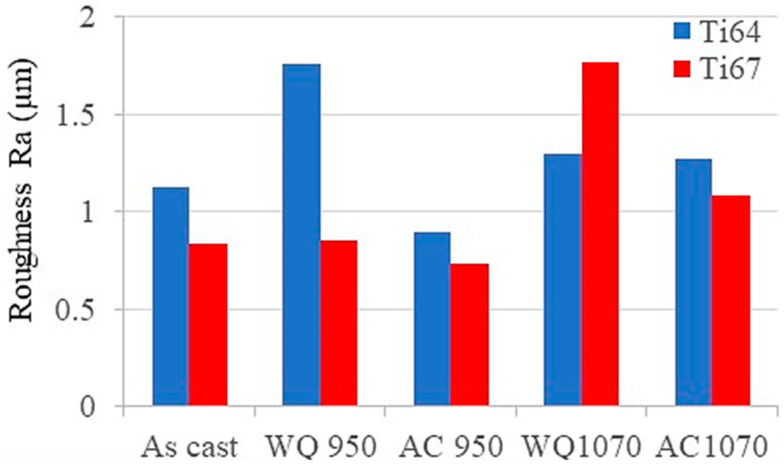
Average surface roughness of the machined holes.

It is well known that the final performance of the machined part is very related to the surface quality after machining. The surface quality of the workpiece can be assessed by measuring the roughness and the chips exerted after drilling. Figure 20 shows the average surface roughness of the machined holes for all the tested workpieces. Generally, it is observed from these measurements that Ti67 alloy shows lower surface roughness and, hence, better surface quality compared to Ti64. This can be partially attributed to the difference between the two alloys in the β-stabilizing element, which is either Nb or V. It has been reported by Zaki et al. [26,27] that Ti64 is more susceptible to oxidation than Ti67. Jiang et al. [41] stated that Nb reduces the solubility and diffusivity of O_2_ in the oxide scale and the base metal. Consequently, the oxide layer in the case of Ti64 will participate in the increased surface roughness. These variations in the microstructures of the tested workpieces with the heat treatment besides the differences in the chemical composition (V/Nb) are expected to show significant differences in the excreted machining chips as well.

**Figure 21:**
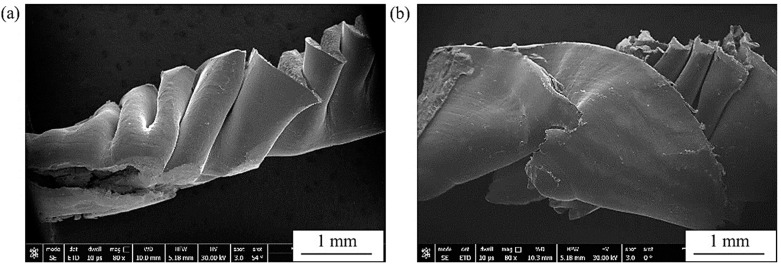
FEM General morphology of the machining chips of cast Ti64 (**a**) and Ti67 (**b**).

**Figure 22:**
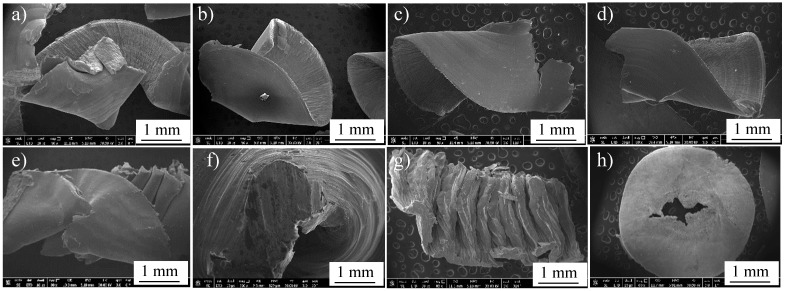
FEM general morphology of the machining chips exerted from Ti64 (**a**–**d**) and Ti67 (**e**–**h**) in WQ950, AC950, WQ1070, and AC1070, respectively.

**Figure 23:**
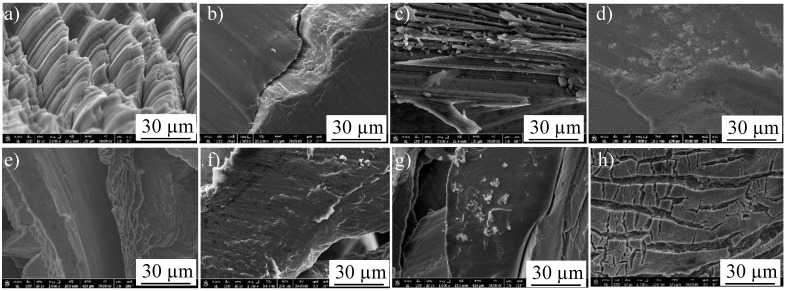
FEM observed morphological features of the machining chips of Ti64 (**a**–**d**) and Ti67 (**e**–**h**) in WQ950, AC950, WQ1070, and AC1070, respectively.

After drilling, machining chips were investigated to reveal the differences in their morphologies among the different workpieces. Figure 21 shows FEM micrographs of the machining chips of cast Ti64 (a) and Ti67 (b). Figure 22 presents the general morphology of the chips obtained from heat-treated conditions, while Figure 23 shows some features observed in different heat-treated workpieces.

Pitches between the adjacent spiral chips and machined surfaces of unbroken chips can be observed in Figure 21. There are also scratches and adhered particles that can be seen in the examples of Figure 23d,f. Remarkable delamination was observed specifically in the case of WQ1070 samples (Figure 23c,g).

To understand the observed differences among all the tested workpieces, the theory of plastic deformation during machining should be clarified. It has been reported by Hussein et al. [42] that a chip segment is first formed ahead of the drilling bit and starts to increase the shear stress. With the progress of machining, the shear stress continues to increase until reaching the critical stage (critical shear stress), where the thermal softening rate is higher than the strain hardening rate. As a result, the phase transformation of α → β occurs, which means that the crystal structure changes from the former (HCP) to the latter (BCC), thus providing a larger number of slip systems and encouraging deformation [43]. This plastic deformation created the white layer and metamorphic layers illustrated in Figure 14 and influenced the morphologies of the exerted chips as well. This shearing action during deformation also resulted in tool wear as described in Section 3.2, which varied depending on the microstructure. The microstructure affects the hardness which, in turn, determines the resistance to the applied shearing stresses during the drilling process. 

**Figure 24:**
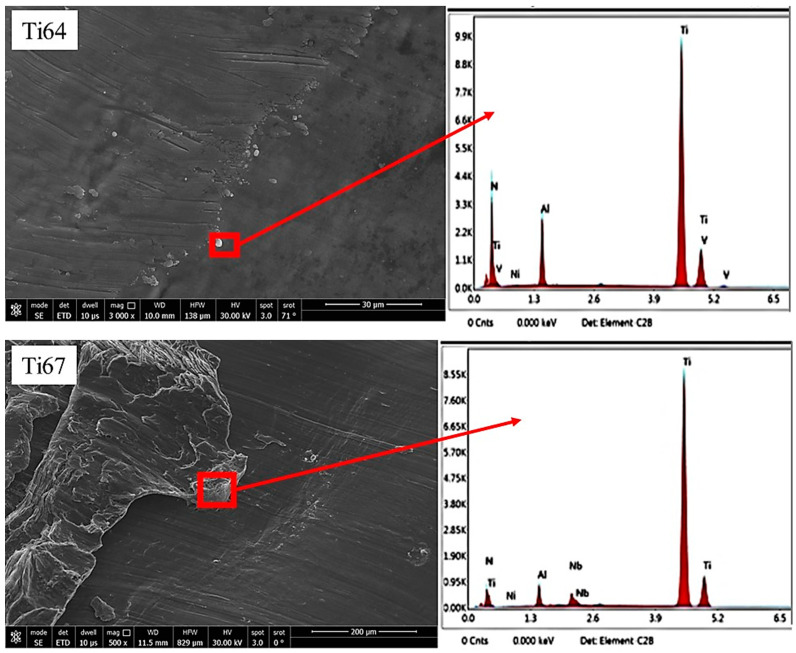
FEM and EDX of the machining chips of cast Ti64 and Ti67 workpieces.

**Figure 25:**
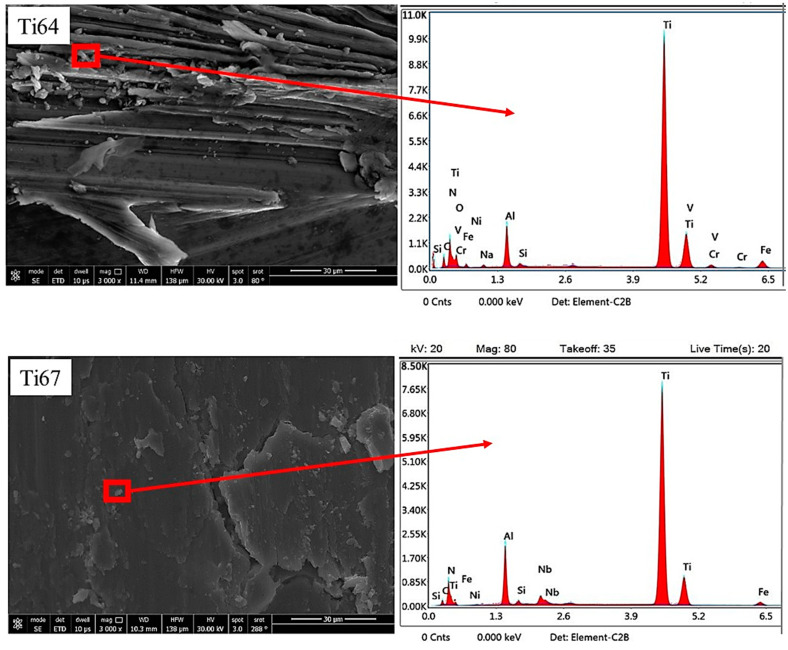
FEM observed morphological features of the machining chips of Ti64 and Ti67 after heat treatment by WQ1070.

To further investigate the relationship between the microstructure of the workpiece and the characteristics of the chip, samples of the chips were analyzed using EDX. Figure 24 shows an analysis of some chips taken from the as-cast Ti64 and Ti67. It is apparent that some particles from the coating were released to the workpiece which was indicated by the presence of N and traces of Ni that came from the bare part of the tool. On the other hand, Figure 25 shows the higher intensity of the coating elements (Ti, Al, and N) and more contaminations from the tool steel (Fe, Cr, Si, etc.) in the case of the heat-treated (WQ1070) samples. This translates to the thicker deformed layer (Table 3) and the higher tool wear (Figure 12) observed in the workpieces (WQ1070) for both of the alloys.

## 4. Conclusions

In the present investigation, Ti-6Al-4V and Ti-6Al-7Nb alloys were prepared by arc melting and received different heat treatment cycles. The samples were machined using computerized drilling tests using TiAlN-coated high-speed steel bits. The influence of the workpieces’ composition and microstructure on their machinability was assessed and is summarized as follows:

1. As-cast Ti67 alloy showed finer grain size and higher transformation temperature (974.7 °C) than Ti64 (950 °C).
2. In the heat-treated Ti64, water quenching from either α/β or β range resulted in the formation of a martensitic structure. The amount of martensite significantly increased upon quenching from the β-phase field.
3. In Ti67, water quenching from α/β phase field (950 °C) did not induce martensite, while some martensite was obtained after quenching from 1070 °C (β-phase range).
4. Generally, water quenching increased the workpieces’ hardness compared to the air- cooling conditions.
5. The workpieces with martensitic structures exhibited higher cutting forces and increased tool wear due to their high hardness.
6. The machined samples with higher hardness resulted in the formation of a thicker sub-machined surface deformation layer (white layer), with some subsurface defects that were frequently observed.
7. In the two alloys, higher surface roughness and burr heights were observed in the workpieces that induced martensite during the heat treatment. Surface roughness in Ti64 workpieces was generally higher compared to Ti67 specimens.
8. Analysis of the as-cast workpieces machining chips showed elemental N indicating the wear of machining bits. Other elements, such as Cr, V, Fe, etc., that came from the uncoated bits, appeared in the water-quenched samples which exhibited high tool wear and thus aggressively attacked the drilling tool.

## Figures and Tables

**Table 1:** Chemical composition of Ti-6Al-4V and Ti-6Al-7Nb alloys.

Element	Al	Nb	V	Ta	Fe	C	O	N	Others	Ti
Ti-6Al-4V	6.1	0.01	4	0	0.1	0.02	0.03	0.01	˂0.4	Bal.
Ti-6Al-7Nb	6.2	6.8	0.01	˂0.05	0.03	˂0.01	0.14	˂0.01	˂0.4	Bal.

**Table 2:** Summary of the heat treatment conditions and alloy codes.

Code	Heat Treatment Conditions	
WQ950	Solution treatment at 950 °C+ WQ	Aging at 550 °C for 4 h
AC950	Solution treatment at 950 °C + AC	
WQ1070	Solution treatment at 1070 °C + WQ	
AC1070	Solution treatment at 1070 °C + AC	

**Table 3:** The average thickness of the deformed sub-machined surface layer, measured in μm using FEM micrographs.

	As-Cast	WQ950	AC950	WQ1070	AC1070
Ti64	6 ± 1.5	3.5 ± 0.58	7.79 ± 1.85	11.95 ± 3.2	9.5 ± 1.7
Ti67	2.9 ± 0.69	10.3 ± 0.54	6.65 ± 1.6	14.3 ± 2.74	8.47 ± 2.9

## Data Availability

Any data not mentioned in the manuscript are available upon request.
